# Postoperative lymphedema after primary total hip arthroplasty: prospective analysis of bikini incision-type direct anterior approach versus established standard approaches

**DOI:** 10.1186/s13018-023-04525-7

**Published:** 2024-01-11

**Authors:** Sylwia Banasiak, Maximilian Hartel, Karl-Heinz Frosch, Josephine Berger-Groch

**Affiliations:** 1 Department of Trauma and Orthopaedic Surgery, University Medical Center Hamburg-Eppendorf, Martinistraße 52, 20246 Hamburg, Germany; 2 Department of Trauma Surgery, Orthopaedics and Sports Traumatology, BG Hospital Hamburg, Bergedorfer Strasse 10, 21033 Hamburg, Germany; 3 Clinic of Trauma and Orthopaedic Surgery, Klinikum Stuttgart, Kriegsbergstr. 60, 70714 Stuttgart, Germany

**Keywords:** DAA, Lymphedema, Swelling, Hypesthesia, Lymphatic drainage

## Abstract

**Background:**

Minimally invasive approaches to the hip joint for total hip arthroplasty such as the DAA (“Direct Anterior Approach with bikini incision”) are increasingly utilized. According to the literature, this approach is more muscle-sparing, results in less postoperative pain, and achieves higher patient satisfaction. The existence of postoperative lymphedema after hip arthroplasty is hardly considered. The aim of this paper is to contribute to the evaluation of the different access methods related to postoperative lymphedema and their functional outcomes.

**Methods:**

This is a prospective non-randomized study at an orthopedic specialist clinic in Northern Europe. The surgeons that performed the arthroplasties are high-volume surgeons in private practice affiliated to the clinic. The study included 188 patients with primary hip arthroplasty in a 1:1 ratio (DAA: standard accesses (posterior, transgluteal, and anterolateral access)). Epidemiologic data, Harris Hip Score, Oxford Hip Score, European Quality of Life 5, and Visual Analog Scale were collected preoperatively on admission day, 3rd and 5th postoperative day, and follow-up after 1 year. Furthermore, the range of motion, gait, and ability to climb stairs, as well as the presence of hypesthesia were assessed. To evaluate the edema situation, both legs were measured on the 3rd and 5th postoperative day. The prescription of manual lymphatic drainage and remaining swelling conditions 1 year postoperatively were recorded.

**Results:**

For each group, 94 patients with a mean age of 61.7 years (DAA 60.7 and standard access 62.6) were included. All but one patient in the DAA group showed postoperative lymphedema (*n*: 93/94; 98.9%). In the standard surgery group, only *n*: 37/94 (39.4%) showed swelling symptoms requiring treatment. After 1 year, lymphedema persisted in 20 patients in the DAA group and 0 patients in the standard-OR group. Hypesthesia at the ventral thigh persisted in 16/94 (= 17%) patients of the DAA group versus 0/94 patients of the standard group after 12 months. Of these 16 cases, 10 had concomitant edema (62.5%). The DAA showed better results than the standard accesses in terms of Oxford Hip Score (*p* < 0.05) and ability to climb stairs (*p* < 0.05). In contrast, the Visual Analog Scale and patient quality of life results showed no significant difference (*p* > 0.05).

**Conclusion:**

The present study demonstrated the increased incidence of postoperative lymphedema in patients operated on via DAA access using a Bikini-type skin incision. In the follow-up, significantly more hypesthesia of the ventral thigh occurred in the DAA group. Otherwise, the DAA proved to be superior to the standard approaches from a functional point of view at short-term follow-up. Future research is needed to compare the horizontally oblique to the longitudinal oblique skin incision technique in direct anterior hip surgery regarding the above-mentioned adverse effects found in this study.

## Introduction

Hip arthroplasty (THA) is one of the most frequently performed and effective surgeries worldwide [1]. In 2019, the average THA implantation rate was 174 per 100.000 inhabitants in the 38 OECD (organization for economic co-operation and development) countries [2]. There are several traditional approaches to the hip joint, such as the posterior, posterolateral, the lateral, or the anterolateral approach in various modifications (standard or minimally invasive) [3]. Detailed data including long-term follow-up (regarding dislocation and revision rates, operation time, loosening, learning curve, costs, blood loss, pain, functional outcome) have been published for all approaches without one approach being entirely superior to the other [4–6]. The choice of the used approach usually depends on the experience of the surgeon. Guidelines with approach selection criteria based on patient-specific factors (anatomy, pathophysiology) are lacking.

The use of more minimally invasive approaches to the hip joint such as the direct anterior approach (DAA) either with a more longitudinal or an oblique bikini incision is increasing [7]. According to the literature, this approach is more muscle-sparing, results in less postoperative pain, and achieves higher patient satisfaction rates [8–11].

Lymphedema is edema with high interstitial protein concentration caused by dysfunctional lymphatic transport capacity [12]. Primary and secondary lymphedema are distinguished. The secondary form can be induced by a lesion of the lymphatic vessels or lymph nodes (i.e., surgery, radiation therapy, chemotherapy, or inflammation/scarring from metastases or filarial diseases) [13]. In clinical practice, secondary lymphedema frequently occurs after orthopedic surgical procedures [12, 14]. The existence of postoperative lymphedema after hip arthroplasty has not been considered for any surgical hip approach to date. A recent study identified preoperative lymphedema as risk factor for complications in primary hip arthroplasty [15]. The aim of this paper is to compare more traditional approaches to the comparably novel DAA with an horizontally oblique bikini-type incision and their relation to postoperative lymphedema and functional outcomes.

## Material and methods

A prospective non-randomized study was performed at a specialist orthopedic clinic in Northern Europe between 2019 and 2021. The surgeons that performed the arthroplasties are high-volume surgeons in private practice affiliated to the clinic. The study included 188 patients with primary hip arthroplasty in a 1:1 ratio (DAA: standard accesses (posterior, transgluteal, and anterolateral access)).

Inclusion criteria for the study were as follows: primary hip osteoarthritis, age 18 years and over, patient's ability to give a written consent, and participation in all examination appointments. Exclusion criteria were: revision surgery, secondary hip osteoarthritis, acute injury elsewhere, acute inflammatory diseases affecting the extremities (e.g., erysipelas, open wounds, hematomas, acute eczema, phlebothrombosis, thrombophlebitis), chronic lip- or lymphedema, malignancies in the affected region, pregnancy, peripheral arterial occlusive disease Grade 3 and 4, cardiorespiratory inability to participate in physical therapy exercise.

Epidemiologic data (age, gender, body mass index, pre-existing conditions), surgical approach, length of surgery, and complication were recorded. Harris Hip Score, Oxford Hip Score, European Quality of Life 5, and Visual Analog Scale were collected preoperatively on admission day, 3rd and 5th postoperative day, and follow-up after 1 year. Furthermore, the range of motion, gait, and ability to climb stairs as well as the presence of hypesthesia, were assessed.

**Fig. 1:**
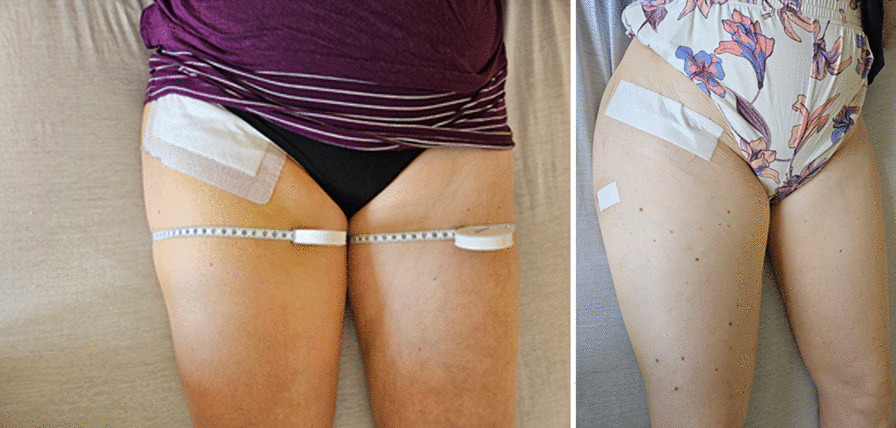
(Left) and (right): two patients with significant swelling at 5th postoperative day after THA via DAA

To evaluate the edema situation, both legs were measured on the 3rd and 5th postoperative day. The swelling was recorded in the supine position by taking measures circumference of the thigh (15cm below spina iliaca anterior superior and 5cm above the patella) and the calf (15 cm below the patella and around the ankle). The measurement position was marked with an indelible marker to ensure that the measurements were continuously recorded on the same part of the lower limb (see Fig. 1). The same staff member completed all measurements. The prescription of manual lymphatic drainage and remaining swelling conditions 1 year postoperatively were recorded.

The local ethics committee approved the present study under file number PV7388-4786-BO-ff.

### Statistics

Descriptive statistics were used to summarize the demographics and clinical characteristics of subjects included. Continuous variables were presented as means and standard deviations. Differences between groups were calculated using the Mann–Whitney U-Test. A *p*-value < 0.05 was deemed significant. Statistical analyses were performed using SPSS statistical software (SPSS version 26.0, Chicago, IL, USA).

The case number calculation was determined with the software G*Power (version 3.1.9.6) [16]. In general, a power of 0.9 (10% error level) was assumed here for two-sided questioning and a mean effect size for the outcome variables of 0.5. This resulted in a necessary total number of cases of *n* = 172 (86 per group) to detect significant differences. To counteract a possible drop-out rate, a total of *n* = 188 (94 per group) were recruited.

## Results

One hundred eighty-eight patients were included in the study. A total of 83 men (age 63 ± 9.8) and 105 women (age 61 ± 8.7) participated in the study without significant age differences between the DAA and standard group (*p* > 0.05). The average BMI was 24.2 ± 2.3 in the male subgroup and 24.3 ± 2.2 in the female subgroup (*p* = 0.45), without a significant difference in the two approach groups (*p* > 0.05) (see Table 1).

**Table 1:** Spreadsheet with epidemiologic data

	Sex	BMI	Age	Hospital stay (d)
*DAA group*				
Male	42	24.6	61.3	
Female	52	23.9	60.3	
*Total:*	94	24.2	60.7	6.2
*Standard group*				
Male	41	24.8	64.5	
Female	53	24.2	61.2	
*Total:*	94	24.6	62.6	9.1
Male	83	24.7	62.9	
Female	105	24.1	60.8	

Ninety-four patients underwent surgery through the DAA and 94 through standard accesses (posterior 32, transgluteal 32, and anterolateral 30). The average length of hospital stay was significantly shorter in the DAA group than in the standard group (DAA 6.2 days ± 1.03, standard 9.1 ± 0.94; *p*-value < 0.05). Regarding Oxford Hip Score, walking distance and climbing stairs significant better results were achieved in the DAA group compared to the standard group. In terms of EQ-5D-3L, Harris Hip Score, length of surgery, and VAS, no significant difference was recorded (*p* > 0.05) (see Table 2).

**Table 2:** Spreadsheet with data comparing standard access (Std) and direct anterior approach (DAA)

	N		Delta-Mean				Delta-SD		F-Test	
	Std	DAA	Std	DAA	t-value	p-value	Std	DAA	*t*-value	*p*-value
EQ-5D-3L	94	94	25.85	25.48	0.36	0.718	6.82	7.30	1.14	0.260
Oxford Hip Score	94	94	20.16	16.34	3.56	**0.000**	7.17	7.54	1.11	0.313
HHS pain	94	94	17.45	18.26	− 0.70	0.487	7.61	8.31	1.19	0.200
HHS ADL	94	94	5.05	4.88	0.40	0.689	2.43	3.33	1.88	**0.001**
HHS walking ability	94	94	6.56	6.53	0.05	0.961	3.97	4.95	1.55	**0.017**
Walking distance 3 to 5 days	94	94	101.6	138.3	− 2.37	**0.001**	72.75	131.5	3.27	**0.000**
Stairs 3 to 5 days	94	94	14.4	33.9	− 5.23	**0.000**	14.63	32.94	5.07	**0.000**
VAS 0 to 3 days	94	94	2.44	2.69	− 1.00	0.318	1.55	1.93	1.55	**0.018**
VAS 3 to 5 days	94	94	1.09	0.69	1.58	0.115	1.04	2.17	4.32	**0.000**
VAS 0 to 5 days	94	94	3.52	3.38	0.69	0.493	1.29	1.46	1.27	0.122

Bold: A p-value < 0.05 was deemed significant

HHS, Harris Hip Score; ADL,  Activity of daily life; VAS,  Visual Analog Scale

Standard surgery had a lower percentage of edema than DAA (standard 39.4% versus DAA 99%). On average, circumferential difference of the thigh was 2.27 cm in the DAA group (♂ 2.14 ± 0.95, ♀ 2.37 ± 1.28, p = 0.315) versus 1.23 cm (♂ 1.32 ± 1.66, ♀ 1.17 ± 1.26, p = 0.556) in the standard group on the 3rd day and 2.89 cm versus 1.27 cm on the 5th day. No significant differences were seen in subgroup analysis regarding age, body mass index, and gender (*p* > 0.05). Twelve months postoperatively, there were still 20 patients in the DAA group with edema in the thigh area. In contrast, there were none in the standard group (*p*-value < 0.05).

After three days, manual lymphatic drainage (MLD) was performed on 37 of 94 patients (≙ 40%) after standard surgery and 93 of 94 patients (≙ 99%) after DAA surgery. MLD is also significantly more frequent after five days after DAA surgery than after standard surgery (55% versus 99%).

At the 12-month follow-up, 16 patients (6 ♂, 10 ♀) in the DAA group (≙ 17%) and 0 patients in the standard group complained of neurological deficits in the sense of tingling, numbness, or burning pain in the ventrolateral thigh (*p*-value < 0.05). Ten of the 16 patients had concomitant persistent swelling of the thigh at the 1-year follow-up.

The complication rate was higher in the standard group in our study with 5 hip dislocations and one infection versus 1 hip dislocation and no infection in the DAA group.

## Discussion

The present prospective study to assess clinical differences between the horizontally oblique DAA and the standard approaches shows that postoperative lymphedema and affections of lateral cutaneous femoral nerve are significantly more frequent in the DAA group than in the standard group. In contrast, the DAA group showed a better early functional outcome (gait range, stair climbing, Oxford Hip Score).

Secondary lymphedema after surgical procedures and trauma to the lower extremity is a well-known clinic phenomenon, but specific incidence data are lacking in the literature. An US-American study described postoperative swelling as the main reason for presentation to an emergency department after THA with 15.6% [17]. This high number illustrates the relevance of the topic and underlines the necessity of perioperative diagnostics and therapy.

The literature provides only scarce information on the topic of lymphedema and hip arthroplasty. In the largest current study on lymphedema and THA, the risk of complications was followed up in 83 patients. It was found that the 5-year infection-free survival rate was 90.3% in patients with lymphedema compared to 97.7% in patients without lymphedema [15]. One case report describes lymphedema as a risk factor for dislocation [18], and another describes how to prepare patients with lymphedema for surgery [19]. Lymphedema was also evaluated as a risk factor for complications in knee and shoulder arthroplasty [20–22]. Studies highlighting problems caused by secondary lymphedema after hip arthroplasty are lacking in the literature.

Currently, the diagnosis is mainly made clinically, but computer-assisted ultrasound imaging is available for further clarification [23, 24]. Evidence-based treatment options have not yet been adequately described. There is literature on taping [25], cryotherapy [26], daily compression bandaging [27], and lymphedema microsurgery [28] after artificial joint replacement. Overall further research is needed in this area.

In this study, 17% of patients operated on via a DAA had persistent affection in the lateral cutaneous femoris nerve after 12 months. Numbers in the literature range from 2 to 31% for this complication at one-year follow-up [11, 29–32]. Spontaneous healing with follow-up periods over 2 years is recorded, and an improvement of dysesthesia as a symptom of LFCN injury was associated with better QOL [31]. So consistent intraoperative sparing of the nerve is important for high postoperative patient satisfaction.

There are heterogeneous results in the literature on patient satisfaction after hip arthroplasty via a DAA. The results range from no difference compared to standard access to significantly better results [33, 34]. There is also evidence that the partly described superior outcomes only prevail in the short term [35, 36]. Consistent with the literature, the present study showed better functional outcomes for the DAA postoperatively in terms of Oxford Hip Score and ability to climb stairs, but no differences in terms of EQ-5D-3L, Harris Hip Score, and VAS.

Over the past 40 years, the rate of dislocations after THA has been significantly reduced. Modifications in the access route are partly blamed for this [37]. The present study showed a slightly lower dislocation rate in the group of patients operated via DAA. However, studies with larger numbers of patients could no longer detect a significant difference [38].

The following limitations need to be mentioned: In rare cases, postoperative lymphoedema may not manifest itself until after 18–24 months. With a follow-up of one year, these patients are missed in this study. A longer follow-up would therefore have been desirable. Another limitation is that a non-randomized study design was chosen. In addition, clinical lymphedema assessment using the thigh circumference is prone to observer bias.

In conclusion, the present study demonstrated the high incidence of postoperative lymphedema in patients operated on via DAA access with the use of a horizontally oblique skin incision. In the follow-up, significantly more hypesthesia of the ventral thigh occurred in the DAA group. Otherwise, the DAA proved to be superior to the standard approaches from a functional point of view. Future research is needed to compare the horizontally oblique to the longitudinal oblique skin incision technique in direct anterior hip surgery with regard to the above-mentioned adverse effects found in this study. The patient's preoperative condition regarding pre-existing swelling tendencies should be considered when selecting the correct access route. Further evaluation is needed, if interventions like preoperative implementation of manual lymphatic drainage, decongestive measures, and screening examinations in neurological terms could improve patient outcome after THA via DAA.

## Data Availability

The dataset used and analyzed during the current study are available from the corresponding author on reasonable request.
